# How often do medical students change career preferences over the course of medical school?

**DOI:** 10.1186/s12909-023-04598-2

**Published:** 2023-08-22

**Authors:** Jean-Sebastien Rachoin, M. Olguta Vilceanu, Natali Franzblau, Sabrina Gordon, Elizabeth Cerceo

**Affiliations:** 1 Division of Hospital Medicine, Department of Medicine, Cooper University Healthcare, Cooper Medical School of Rowan University, Suite 223 Dorrance Bldg. One Cooper Plaza, Camden, NJ 08103 USA; 2 Department of Public Relations and Advertising, Rowan University, Glassboro, NJ USA; 3 Department of Obstetrics and Gynecology, Cooper University Healthcare, Cooper Medical School of Rowan University, Camden, NJ USA; 4 Department of Medicine, Cooper University Healthcare, Camden, NJ USA

**Keywords:** Medical students, Medical school, Residency, Career, Choice

## Abstract

**Introduction:**

During the preclinical years, students typically do not have extensive exposure to clinical medicine. When they begin their clinical rotations, usually in the third year, the majority of the time is spent on core rotations with limited experience in other fields of medicine. Students then must decide on their careers early in their fourth year. We aimed to analyze how often medical students change their career preferences between the end of their second and their fourth year.

**Methods:**

We conducted a retrospective, cohort study using the American Association of Medical Colleges Year 2 Questionnaire (Y2Q) and Graduating Questionnaire (GQ) from 2016 to 2020.

**Results:**

20,408 students answered both surveys, but 2,165 had missing values on the career choice question and were excluded. Of the remaining students, 10,233 (56%) changed their career choice between the Y2 and GQ surveys. Fields into which students preferentially switched by the GQ survey included anesthesia, dermatology, ENT, family medicine, OB/GYN, pathology, PM&R, psychiatry, radiology, urology, and vascular surgery. Many characteristics, including future salary, the competitiveness of the field, and the importance of work-life balance, were significantly associated with a higher likelihood of changing career choices. On the other hand, having a mentor and the specialty content were associated with a lower likelihood of change.

**Conclusion:**

A majority of students switched their career preferences from the Y2Q to the GQ. Additional research should be focused on curricular design that optimizes student satisfaction with career decisions. This may include early integration of a variety of specialties.

## Introduction

Many medical students experience a great deal of stress in career decision-making with 15% remaining still undecided about their preferred career choice after graduation [1–3]. A variety of factors contribute to medical students’ career paths including mentors, research in a given field, experiences prior to medical school, specialty-specific factors, and interest in the pathophysiology of a specialty. Clinical exposure, while not the only factor, can be an important one [4, 5]. However, students often experience the various fields in the fourth year, after they must select their future residency.

While the educational structure of US medical schools is evolving, the traditional format has been two preclinical years, spent mostly in classroom settings where students learn basic sciences and foundations of medicine, followed by two clinical years, where students are exposed to the different clinical specialties and subspecialties on a rotational basis. During the third year, they spend 12 months between required rotations (such as surgery or general medicine) and some elective ones (such as pathology, radiology, or specific subspecialty). At the beginning of their fourth year, they have a very short window (less than 3 months) to submit their applications for a residency position.

Given the tight timeline between exposure to a field and decisions on one’s career paths, there may be many students who are still unsure of career preferences at the end of the third year. Even among core rotations, students may have a difficult time deciding. When there are many fields in which students do not get a chance to participate, the likelihood may increase that some students will be excluded from a potentially rewarding career or at least led to increased stress around career decisions for a perceived irreversible choice [6]. Additionally, students who decide on more competitive fields later in their medical school training may be at a disadvantage as they may have missed opportunities to network and express interest, do specific rotations, and be involved with research or scholarly activity.

Studies that looked at career paths for medical students have analyzed factors that influenced their choices and the characteristics that were associated with them [7–11]. Little is known however about how medical students’ choices may shift over the course of their medical school training and the factors associated with a potential change in career orientation.

## Methods

We conducted an analysis of survey responses from the Year Two Questionnaire (Y2Q) and the Graduating Questionnaire (GQ) survey administered by the Association of American Medical Colleges (AAMC) for the years 2016–2020. This data was obtained from and used with the permission of the AAMC.

### Y2 questionnaire and GQ survey

The Y2 and GQ surveys are administered to all active second-year medical students (Y2Q) and graduating students (GQ). They include questions regarding future career plans, learning climate (Y2) and general medical education, readiness for residency, and career intentions (GQ) among others [12, 13]. These surveys are administered at 135 medical schools in the USA and their results are used to improve the quality and medical students’ experience. The surveys directly ask students about the relative importance of specific factors in career decision-making including the following: competitiveness of the specialty, their level of educational debt and income expectations, influence of mentors, having the option to do fellowship training and length of training, family expectations and family planning, work/life balance, and alignment with personality, interests, and skills.

### Student sample and variables

We included students who answered both the Y2Q and GQ surveys between the years 2016 and 2020. We recorded answers to the survey questions including information about student loans and debt. The following self-reported demographic variables: age range, gender, sexual orientation, and race were also included. For the variable race, we considered students with more than one reported race as multiracial. While the Surveys themselves do not specifically ask demographic questions, they connect with other AAMC applications that do. We analyzed responses to questions regarding preferred specialties and factors that influenced their career choice.

### Statistical analyses

As stated above, we only kept in the study students who answered both surveys. We analyzed demographic variables, survey responses, present continuous data as mean (standard deviation) and categorical variable as percentages. We used univariate analyses (Chi-square and T-test) and performed a multivariable regression analysis to assess interdependence of factors regarding the propensity to change the choice of career. We used a forward conditional methodology and considered variables to have significant association with the outcome of interest if p < 0.05. Forward conditional regression analysis is a technique that allows for stratification and matching and is best suited for observational studies [14]. With this technique, variables are added sequentially starting with the most significant and then kept or removed if the p-value remains significant. The final model will only include significant variables. All the variables with the exception of amount of debt were categorical. All analyses were done using SPSS, IBM 28.0 software, Chicago, Illinois, USA.

## Results

There were 38,543 surveys for Y2Q and 49,455 for GQ but only 20,408 students answered both surveys. 2,165 students had missing values for the questions related to career choice and were removed from further analysis. Demographic questions from the GQ survey are presented in Table 1.

**Table 1:** Demographic variables

Variable	Count
Gender	
Female	10,814 (53%)
Male	9,594 (47%)
Sexual Orientation	
Bisexual	719 (3.5%)
Gay/lesbian	861 (4.2%)
Heterosexual	17,612(86.3%)
Missing	1,216 (6%)
Age	
Under 24	57 (0.3%)
24 to 26	8714 (42.7%)
27 to 29	8534 (41.8%)
30 to 32	2014 (9.9%)
33 or older	1089 (5.3%)
Race	
White	12,304 (60.3%)
Asian	4025 (19.7%)
Hispanic	877 (4.3%)
Black	1077 (5.3%)
Other	385 (1.9%)
Multiracial	1666 (8.2%)
Missing	74 (0.4%)
Total Debt (per $1,000)	155.5(126)

### Choice of specialty in Y2 and GQ

8,010 (44%) students chose the same career on both the Y2Q and GQ surveys and 10,233 (56%) changed their career choice. The most chosen specialty was internal medicine followed by pediatrics and emergency medicine on both the Y2 and GQ survey. Surgery was the fourth choice on the Y2Q survey but dropped to the sixth choice on the GQ survey, behind Family Medicine and OB-GYN. (Table 2)

**Table 2:** Specialty choice Y2 and GQ

	Total in Y2	Total in GQ	Students that did not change Y2 = GQ	Students with change in interest	Change in interest as percent
Anesthesia	453	1,078	265 (58.5%)	625	138.0%
Dermatology	278	334	130 (46.8%)	56	20.2%
Emergency Medicine	1,915	1,809	1,074 (56.1%)	-106	-5.5%
Family medicine	1,077	1,643	701 (65.1%)	566	52.6%
Genetics	19	15	6 (31.6%)	-4	-21.0%
Internal Medicine	3,185	3,427	1,708 (53.6%)	242	7.5%
Neurosurgery	241	178	102 (42.3%)	-63	-26.2%
Neurology	452	439	177 (39.2%)	-13	-2.8%
OB-GYN	985	1,322	518 (52.6%)	337	34.2%
Ophthalmology	373	375	195 (52.3%)	2	0.6%
Orthopedics	923	694	464 (50.3%)	-229	-24.8%
ENT	286	365	129 (45%)	79	27.3%
Pathology	125	148	67 (53.6%)	23	18.4%
Pediatrics	1,943	1,927	1,090 (56.1%)	-16	-0.8%
PM&R	139	207	65 (46.8%)	68	49.0%
Plastics	170	159	57 (33.5%)	-11	-6.6%
Psychiatry	386	1,051	273 (70.7%)	665	172.2%
Radiology	413	734	227 (55%)	321	77.8%
Radiation oncology	124	108	36 (29%)	-16	-13.0%
Surgery	1438	1,185	482 (33.5%)	-253	-17.6%
Vascular surgery	40	53	11 (27.5%)	13	32.5%
Thoracic surgery	124	101	38 (30.6%)	-23	-18.7%
Urology	183	306	79 (43.2%)	123	67.3%
Med-Peds	526	381	92 (17.5%)	-145	-27.5%

The specialties that maintained the most consistent choices among students were psychiatry (263, 70.7%), family medicine (701, 65.1%), anesthesia (265, 58.5%), emergency medicine (1,074, 56.1%) and pediatrics (1,090, 56.1%).

Between Y2Q and GQ, numerous students changed their career choice. The specialty attracting the most students by the GQ was psychiatry (+ 665, 172%), anesthesia (+ 625, 138%) and radiology (+ 321, 77.8%). The specialties that saw the largest drop were med/peds (-145, -27.5%), neurosurgery (-63, -26.2%) and orthopedics (-229, -24.8%).

### Comparison between students with similar vs. change in career choice

Gender, sexual orientation, and age did not affect changing residency choices but race did, with more White students staying in the same selected field and more minorities (Asian, Black, Hispanic, multiracial, other) switching to another field. **(**Table 3**)** There were important differences in factors that affected the students’ career decisions as shown in Table 3. Options to do a fellowship, the plan to enter a loan forgiveness program, and the amount of debt were not statistically significantly associated with the stability of students’ career decisions.

**Table 3:** Comparison between students who changed career choice or not

	Same	Changed	P value
**Number of students**	8,010	10,233	
**Gender Female**	4224 (52.7%)	5492 (53.7%)	0.209
**Sexual orientation**			
Bisexual	283 (3.6%)	397 (4%)	
Gay/Lesbian	330(4.2%)	467 (4.7%)	0.134
Heterosexual	7296 (92.2%)	9176 (91.4%)	
**Age**			
< 24	22 (9.3%)	29 (0.3%)	
24–26	3,500 (43.7%)	4,309 (42.1%)	
27–29	3,292 (41.1%)	4,329 (42.3%)	0.143
30–32	760 (9.5%)	1,038 (10.1%)	
33 or older	436 (5.4%)	528 (5.2%)	
**Race**			
White	5,065 (63.2%)	6,153 (60.1%)	
Asian	1,456 (18.2%)	2,035 (19.9%)	
Hispanic	320 (4%)	431 (4.2%)	0.004
Black	377 (4.7%)	535 (5.2%)	
Other	133 (1.7%)	192 (1.9%)	
Multiracial	633 (7.9%)	848 (8.3%)	
Plan to enter Loan forgiveness program	2717 (45.2%)	3588(44.4%)	0.303
Debt Total (in $1,000)	154(127)	157(126)	0.134
**Factors (Moderate/major influence)**			
Competitiveness of specialty	2,661 (33.3%)	4,707 (40.9%)	< 0.001
Impact of Debt	1,485 (18.6%)	2,593 (22.6%)	< 0.001
Influence of role models	6,601 (82.6%)	9,210 (80.1%)	< 0.001
Possibility to do a fellowship	4,810 (60.2%)	7,030 (61.2%)	0.156
Importance of compensation	3,457 (43.3%)	5,562 (48.4%)	< 0.001
Length of training	3,245 (40.6%)	5,035 (43.8%)	< 0.001
Family expectations	2,153 (27%)	3,365 (29.3%)	< 0.001
Family Plan	4,352 (54.4%)	6,651 (57.9%)	< 0.001
Importance of work/life Balance	6,017 (75.4%)	9,034 (78.6%)	< 0.001
Content of specialty	7,882 (98.7%)	11,264 (98.1%)	< 0.001
Personality fit	7905 (98.9%)	11,334(98.6%)	< 0.001

Multivariable regression analysis demonstrated an association with higher likelihood of changing specialties among the following factors: the competitive nature of the specialty, the compensation, and work/life balance. Influence of role models and content of specialty was associated with a lower likelihood of change. **(**Table 4**)**

**Table 4:** Factors associated with Change in career choice:

Factor	OR	P value
Competitiveness of specialty	1.34[1.24–1.44]	< 0.001
Importance of work/life Balance	1.14[1.05–1.24]	0.002
Importance of compensation	1.13[1.05–1.22]	< 0.001
Influence of role models	0.86[0.78–0.94]	< 0.001
Content of specialty	0.63[0.47–0.86]	0.003

## Discussion

Medical students are asked to learn a vast amount of material in their preclinical years in anticipation of clinical rotations where they are expected to decide their area of practice for the next several decades. However, clinical and theoretical practice can often differ greatly. Medical students may enjoy studying the pathophysiology of a given process but not the day-to-day practice associated with it. Certainly, the opposite can be true as well; a subject that is not especially appealing in undergraduate medical education may be very satisfying if considering the affected patient population or procedures they can perform. Thus, if there is no concomitant clinical exposure and the material is considered in a vacuum, the students may misunderstand the nuances of a given field.

Between the second and the fourth year of medical school in the USA, almost 60% of students change their mind regarding their intended field of practice. While there may be many reasons for this switch, students may benefit from broader exposure to various clinical fields in their preclinical years and third year. Given the current structure of most medical school curricula with limited clinical interactions in the first two years, many students may not have sufficient experience to make an informed decision about their future career choices. Students essentially have until the end of their third year and perhaps beginning of the fourth year to decide on the path for the rest of their professional lives.

An important question raised by this study is whether students are switching their career decisions after exposure to clinical practice as a whole or if there is benefit to a more diverse set of electives to experience specific fields. Given trends to truncate medical training, there is the potential to exacerbate this phenomenon, allowing for even less time and clinical exposure on which to base career decisions. While the study design does not allow for answers to such nuanced questions, it identifies a need for flexibility especially among the 15% of medical students who remain undecided at graduation [15].

Models of exposing students to the clinical environment in the first and second year may be useful to mitigate this uncertainty but associations have not been researched [16–19]. The modes of early integration of clinical experiences is also not uniform. Some schools may employ brief, week-long burst weeks rotating through different specialties in the hospital or ambulatory settings while other may use a longitudinal clinic experience.

A potential solution is interspersing exposure to less common rotations among the core rotations during the third year. Certainly, the knowledge gained in core rotations cannot be supplanted but understanding the details of a given field may be as important to students, as they map out their career trajectory. Because the third year is particularly weighty with students getting the bulk of their clinical exposure then, it could be argued that there is no time to include other specialties into the schedule and that students often have a few weeks of electives that could be used to explore non-core fields. However, students do not know what they do not know. Many may have never considered pain medicine, anesthesiology, or vascular surgery and so it would not cross their minds to carve out elective time for some specialties. For example, low visibility of geriatrics in the curriculum has been associated with low interest as a specialty [20]. Mandatory participation in a broad range of elective experiences interspersed throughout the third year would accomplish this with the added benefit of building knowledge and understanding for colleagues in other fields of medicine.

Another option that could be explored would be a transitional-type intern year for undecided students that would allow exploration of various fields with broad clinical exposure. Transitional years have already been employed in surgical specialties and others. Such a structure could be flexibly designed with various components that would reflect the reality of practice in the fields. The year, recognized as a valid intern year, could be applied to whatever area the residents then chose to pursue, affording greater career goal alignment and potentially greater future career satisfaction. The challenge would be in restructuring and allocating subsequent residency slots after the first year. However, this inconvenience may be offset by decreased later attrition and greater job satisfaction.

Other reasons for putting intentionality behind thoughtful career development design relates to the student populations most at risk. (Table 3) More minorities (Asian, Black, Hispanic, multiracial, other) switched anticipated fields of practice from their second year (Y2) to their fourth year (GQ). This could be due to differences in exposure to medical fields earlier in life, from differing expectations or perceptions of the field, or other factors not yet explored. Targeted interventions including earlier diverse career exposure may be especially helpful for these groups or for any student who self-identifies as undecided. Resources could then be diverted appropriately as there will be students who have determined their career path early on.

Schools can facilitate finding mentors since they can guide, provide feedback, and manage expectations and are associated with a lower likelihood of changing fields. Schools should also be transparent with factors such as future salary and the perceived competitiveness of fields. Individual and personality factors may affect perceptions around the duration of training and demands of the field as well as family planning. The change in career choice may also be a practical one as exam scores and clinical performance may preclude access to more competitive fields for some students. Thus, resources focused on supporting students through appropriate career decisions may better align reported interests.

The application demands of competitive fields put those students who do not decide early on that specialty at a disadvantage. There are residencies that will place greater emphasis on research output and, if students decide in their third year to do a certain specialty, they may not have time to produce the scholarly output that would make them a competitive candidate. This may starve certain fields of needed personnel and direct students to other fields where they may not find as much professional fulfillment.

There have not been systematic assessments of the cumulative impacts of those medical schools with early clinical exposure nor specific effects on career decisions. The types of early clinical experiences is diverse, ranging from training and experience as emergency medical technicians, health systems navigators, and longitudinal patient clinics [21]. Among 69 longitudinal clinical programs, skill development increased among the pre-clinical students but impacts on career decisions were not explored [22]. Individual programs integrating early clinical experiences to promote careers in primary care have led to increased student satisfaction though may not have realized the career development goals that were anticipated [23]. Thus schools must engage in active curriculum management and monitoring along with assessment of student outcomes to align clinical content [24].

To be sure, students who enter any of the various specialties can find personal and professional fulfillment as they interact with patients and pursue their intellectual curiosity. Professional satisfaction does not necessarily equate with one sole field and people could find joy in many areas of medicine. Additionally, some physicians have changed careers after initial residency or fellowship but this requires many more years of training and can lead to financial and emotional stress. A less modifiable factor in career choice is that there are remarkable developmental changes occurring in young people especially under 25 years of age that could impact decision-making in various directions. Other limitations in the research include those imposed by aggregate data accrued from surveys. While it allows access to broad swaths of the medical students, it limits conclusions that can be drawn and flattens the population. Focus groups, more nuanced questions, or free text responses may create a more holistic picture of motivations behind career planning. Individual medical schools may be well-served to use their own data to assess how often students are making career changes and then modifying their unique curriculum to the circumstances. Another important consideration is that the US is currently facing a crisis in primary care and one could argue that a curriculum that emphasizes primary care responds to current needs more than one that accentuates further sub-specialization. An integrated curriculum can accomplish both ends though, exposing students to the many fields in medicine while simultaneously showing the benefits of a career in primary care. Obviously, many additional societal, workload, and reimbursement changes in primary care would also need to be instituted to attract more students to the field but early emphasis on the importance of primary care starting with medical school curricula may support a shift in medical priorities.

Such restructuring of the clinical years requires and a thoughtful, individualized approach, leaning on the strengths of various medical schools and health systems. One-size-fits-all programmatic development will minimize nuance and not meet the needs of students and so adaptability must be the hallmark of clinical integration. Successful integration may expand the horizons of students and optimize career alignment for many. Future medical education research should continue to promote means to enhance and augment clinical experience throughout the undergraduate medical education continuum.

## Conclusion

A majority of students switched their career preference from the Y2Q to the GQ. Many characteristics, including future salary, the competitiveness of the field, and the importance of work-life balance, were significantly associated with a higher likelihood of changing career choice. Having a mentor and the specialty content were associated with a lower likelihood of change. Curricula focused on early integration of a variety of specialties should be designed with a goal of aligning with student interests and needs.

## Data Availability

The dataset used for this study was obtained and used with the permission of the American Association of Medical colleges (AAMC) for the sole purpose of this research project. The AAMC reviewed and approved the results and manuscript. The data can be obtained from the AAMC after a written request. We are not permitted to make it available publicly. David Matthew from the AAMC The AAMC can be contacted to obtain the dataset. dmatthew@aamc.org.
